# A Role for Iodide and Thyroglobulin in Modulating the Function of Human Immune Cells

**DOI:** 10.3389/fimmu.2017.01573

**Published:** 2017-11-15

**Authors:** Mahmood Y. Bilal, Svetlana Dambaeva, Joanne Kwak-Kim, Alice Gilman-Sachs, Kenneth D. Beaman

**Affiliations:** ^1^Clinical Immunology Laboratory, Rosalind Franklin University of Medicine and Science, North Chicago, IL, United States; ^2^Department of Microbiology and Immunology, Rosalind Franklin University of Medicine and Science, North Chicago, IL, United States; ^3^Department of Obstetrics and Gynecology, Rosalind Franklin University Health System, Vernon Hills, IL, United States

**Keywords:** iodine, iodine deficiency, RNAseq, nutritional immunology, thyroid hormones, thyroglobulin, NIS, pendrin

## Abstract

Iodine is an essential element required for the function of all organ systems. Although the importance of iodine in thyroid hormone synthesis and reproduction is well known, its direct effects on the immune system are elusive. Human leukocytes expressed mRNA of iodide transporters (NIS and PENDRIN) and thyroid-related proteins [thyroglobulin (TG) and thyroid peroxidase (TPO)]. The mRNA levels of PENDRIN and TPO were increased whereas TG transcripts were decreased post leukocyte activation. Flow cytometric analysis revealed that both PENDRIN and NIS were expressed on the surface of leukocyte subsets with the highest expression occurring on monocytes and granulocytes. Treatment of leukocytes with sodium iodide (NaI) resulted in significant changes in immunity-related transcriptome with an emphasis on increased chemokine expression as probed with targeted RNASeq. Similarly, treatment of leukocytes with NaI or Lugol’s iodine induced increased protein production of both pro- and anti-inflammatory cytokines. These alterations were not attributed to iodide-induced *de novo* thyroid hormone synthesis. However, upon incubation with thyroid-derived TG, primary human leukocytes but not Jurkat T cells released thyroxine and triiodothyronine indicating that immune cells could potentially influence thyroid hormone balance. Overall, our studies reveal the novel network between human immune cells and thyroid-related molecules and highlight the importance of iodine in regulating the function of human immune cells.

## Introduction

Iodine is an essential mineral required for the biosynthesis of thyroid hormones and subsequent proper function of metabolic pathways of all body organs (1). Disorders stemming from iodine deficiency or insufficiency are a worldwide issue affecting approximately two billion people including school-aged children (2, 3). The requirement for sufficient iodine levels encompasses all stages of life (2, 4). First, increased iodine levels are required during pregnancy, and reduced amounts lead to miscarriages and reproductive failures (5–8). This is in-part due to the role of iodine-derived thyroid hormones, thyroxine (T_4_), and triiodothyronine (T_3_), for optimal fetal brain development (9, 10). Second, congenital hypothyroidism, defined by reduced thyroid hormones leading to stunted mental and physical development during early childhood, is caused by insufficient iodine intakes (2–4, 11). In adults, non-optimal iodine intake causes hypothyroidism and goiter formation that could be reversed with increased iodine intakes or supplementation (2, 4, 12, 13). Therefore, iodine is required at all stages of life, and its decreased uptake will lead to potentially life threatening conditions and/or severe reduction of quality of life.

Production of thyroid hormones begins with iodide transportation into thyroid follicular cells in the thyroid gland *via* the sodium iodide symporter (NIS) (14, 15). Iodide molecules are then shuttled through another receptor, PENDRIN, into the thyroid colloid/lumen. Membrane bound thyroid peroxidase (TPO) enzymes oxidize iodide into iodine, a reaction needed for the eventual conjugation or organification of iodine into tyrosyl compounds present on the large dimeric protein thyroglobulin (TG). These biochemical pathways are the source of iodinated tyrosines that eventually form thyroid hormones T_4_ and T_3_. T_4_ forms 80–90% of total biologically active hormones made by the thyroid and are stored in TG (15–17). TG is endocytosed by follicular cells and undergoes proteolytic degradation thereby releasing mostly T_4_ and relatively low amounts of T_3_ into peripheral blood. The activity of tissue bound deiodinase enzymes converts T_4_ into the more biologically active hormone T_3_ (15, 18). Ultimately, thyroid hormones affect the metabolic processes of cells, which include gluconeogenesis, glycogenolysis, thermogenesis, and protein metabolism (15).

Interestingly, thyroid hormones can directly affect multiple branches of the immune system by enhancing dendritic cell antitumor immunity, B cell differentiation, phagocytosis, natural killer cytotoxicity, inducing higher expression of cytokines, and increasing the frequency of T cell memory cells (19–23). The effects of thyroid hormones on immune cells are due in-part through activation of protein kinase C signaling (20). Furthermore, immune cells are able to produce TSH and utilize TSH to increase T_3_ levels (24, 25). Thus, far no significant sources of *de novo* T_4_ that could affect tissue or blood hormone levels have been identified other than the thyroid gland. However, intriguing findings by Nagao et al. and others demonstrated the likelihood of extrathyroidal T_4_ synthesis in thyroidectomized rats (26, 27). Others have shown the presence of low intracellular levels of thyroxine in cardiomyocytes utilizing radioactive iodide ^125^I, and the presence of “thyroxine-like” compounds in ^131^I-pulsed leukocytes (28, 29). The prospect of extrathyroidal T_4_ production is strengthened by experiments demonstrating the presence of iodinated tyrosines and thyroid biosynthesis machinery (NIS, TG, and TPO) in multiple tissues including the endometrium, placenta, mammary glands, thymus, testis, liver, and kidneys (30–34). These studies reflect the potential for iodide influx into these tissues. Overall, sources of extrathyroidal thyroxine remain elusive.

The direct effects of inorganic iodine or iodide on cellular activity of immune cells, outside of thyroid hormones, remain relatively unexplored. Evidence for possible direct role for iodine on immune cells was demonstrated by Marani et al. wherein school children deficient in iodine had reduced immune responses despite normal thyroid hormone levels (35–37). Further studies on human breast cancer cells demonstrated the effects of Lugol’s solution, composed of molecular iodine (I_2_) and potassium iodide (KI), on the transcriptional activity of these cells (38). Other iodine concentrating tissues have been identified including ovaries, salivary glands, and the thymus (39, 40). Similarly, a 1971 study by Stolc showed that ^131^I-pulsed leukocytes could concentrate iodide intracellularly (28). Xiaoyi et al. studied the cytotoxic effects of molecular iodine in murine immune cells and found, increased lymphocyte survival, slightly reduced CD4/CD8 ratios, and increased IFNγ/IL4 ratio upon activation. Nonetheless, the effects and mechanisms of iodide, as well as molecular iodine, on the function immune cells remains ill explored. The immune system not only protects against foreign pathogens, tumors, and autoimmune responses but it can also modulate and provide a growth milieu during tissue repair and pregnancy through the production of growth factors and angiogenesis (41–43). During the stages of pregnancy, the balance between pro- and anti-inflammatory factors needs to be actively balanced through production of multiple cytokines and immune agents (41, 44).

Due to the importance of iodine and thyroid hormones in pregnancy and the modulatory roles of the immune system on this process, we sought to explore the interplay between thyroid-related molecules and the immune system. In this study, we have examined the effects of inorganic iodine/iodide on cellular function and assessed if immune cells could secrete thyroid hormones. To this end, we have analyzed the expression of iodide transporters in normal donor peripheral immune cells and determined if iodide induces functional changes in the activity these cells. Our studies show a pronounced iodide-induced transcriptional and cytokine response by human peripheral blood leukocytes that were not attributed to new thyroid hormone synthesis. Interestingly, substantial amounts of thyroid hormones were released upon incubation of leukocytes with thyroid-derived TG. Altogether, these observations demonstrate a novel insight on the effects of iodide on human immune cells and highlight leukocytes as a potential source for T_4_ in local tissues and peripheral blood.

## Materials and Methods

### Purification and Culture of Human Leukocytes and Jurkat E6.1 Cell Line

Human leukocytes were obtained from blood samples drawn in sodium heparin where donors have consented for blood donation. The consent and documentation process associated with these donors were approved by the IRB for Rosalind Franklin University of Medicine and Science. Leukocytes were extracted with standard Ficoll-Paque method. The cells were cultured at 37°C and 5% CO_2_ in complete RPMI 1640 (RPMI medium supplemented with 10% FBS, 50 U/mL penicillin, 50 µg/mL streptomycin, and 2 mM l-glutamine) (Gibco). The E6.1 Jurkat T cell line was acquired directly from ATCC (TIB-152) and cultured at 37°C and 5% CO_2_ in complete RPMI medium.

For cell counts or viability assays, 5 × 10^6^ leukocytes were seeded into 1 mL complete RPMI media with 1 mM NaI (383112, Sigma) or PBS (control) for 3 days. Cells were counted using BIO-RAD TC20 automated cell counter. Viability was determined with standard Trypan Blue (T8154, Sigma) exclusion assay analyzed by TC20 cell counter software.

### RNA Extraction from Leukocytes

5 × 10^6^ leukocytes were suspended in 1 mL complete RPMI media, and then left unstimulated or activated with PMA (25 ng/mL) and ionomycin (1 µM) for 18 h and then total RNA was extracted from leukocytes utilizing Qiagen’s mini RNeazy kits. For targeted RNASeq, leukocytes were incubated with or without 1 mM NaI or PBS (control) for 48 h.

### Reverse Transcription PCR/Quantitative PCR (qPCR)

Reverse transcription was performed with 400 ng of total RNA using transcriptor first strand cDNA system (Roche). 4 µL of cDNA was amplified for 35 cycles with Amplitaq DNA polymerase kit (Life Technologies). The following primers were used for PCR amplification with all primers listed in the 5′ → 3′ direction (NIS F 287 bp: CTCTTCATGCCCGTCTTCTAC, NIS R: GACAACCCAGAAGCCACTTA), (PENDRIN F 320 bp: TCCTGTCGGATATGGTCTCTAC, PENDRIN R: GATCTGCCAAGTACCTCACTATG), (TPO F 274 bp: GGAAGCAGATGAAGGCTCTG, TPO R: AGTGCACAAAGTCCCCATTC), and (GAPDH F 440 bp: ACATCATCCCTGCCTCTACT, GAPDH R: CTCTCTTCCTCTTGTGCTCTTG).

For quantitative real-time PCR, 2 µL cDNA was amplified with TaqMan Fast Advanced Master Mix (Applied Biosystems) and read with StepOnePlus real-time PCR instrument (Applied Biosystems). Validated TaqMan primer mix was obtained from Invitrogen as follows: NIS: Hs00950365_m1, PENDRIN: Hs01070627_m1, TG: Hs00174974_m1, and B2M: Hs00187842_m1. Gene expression was normalized to internal B2M amplification.

### Targeted RNASeq *via* Next-Generation Sequencing (NGS)

Next-generation sequencing Library preparation was performed per Qiagen’s targeted RNASeq *Human Inflammation & Immunity Transcriptome* panels containing probes for 475 genes. Targeted RNASeq is more quantitative and specific than conventional qPCR due to specific transcript sequencing and the unique molecular barcoding of each transcript, detected with bioinformatics, before PCR amplification. In this way, molecular barcoding bypasses PCR bias that could affect conventional qPCR results resulting from inefficient primers and/or poor sample preparation. Briefly, cDNA was made from 400 ng of leukocyte RNA, and then unique molecular 12 nucleotide tags were incorporated into a total of 20 ng cDNA *via* gene specific primer extension. After PCR purification using magnetic beads, the barcoded cDNA was amplified utilizing gene specific primers. The purified DNA was again amplified through a second PCR reaction to insert index sequences that are unique to each sample. This step allows the combination of multiple samples in one tube for subsequent sequencing. The completed library was loaded into Illumina’s reagent cartridge (150 cycle v3) with a standard flow cell and custom sequencing primer provided by Qiagen. NGS was performed on Illumina’s MiSeq instrument per manufacturer’s recommendations. Sequencing quality controls, including cluster density, total reads, and percent reads reaching Q30, were all within optimal ranges provided by Illumina. In addition, secondary quality controls provided by Qiagen’s targeted RNASeq software that reads and quantifies the sequencing files were all within acceptable ranges. The FASTQ files obtained from the sequencing runs were uploaded to Qiagen’s *GeneRead DNAseq variant calling service*. The data were then exported into a format that provides the total unique molecular barcode sequencing reads per gene. All reads/samples were normalized to 10 internal control housekeeping genes after screening negative for genomic DNA contamination. Statistical analysis was then performed on normalized data that were quantified as fold change compared with the associated controls.

### Enzyme-Linked Immunosorbent Assay (ELISA)

5 × 10^6^ leukocytes were incubated in complete RPMI media with PBS (control) or 1 mM NaI for 72 h, and then the supernatant was collected. Alternatively, the cells were treated with 500 µM of Lugol’s iodine solution (32922, Sigma). Cytokines were quantified utilizing Invitrogen’s ELISA kits per manufacturer’s recommended protocol (Invitrogen IFNγ: 88-7316; IL6: 88-7066; IL8-CXCL8: 88-8086; IL10: 88-7106; CCL2: 88-7399). ELISA plates were read using a spectrophotometric plate reader at a wavelength of 450 nm.

### Flow Cytometry

5 × 10^6^ leukocytes were washed in PBS and then incubated in 500 µL PBS with 10% goat serum (S-1000, Vector Laboratories) for 30 min to block non-specific binding. The cells were resuspended in 100 µL PBS with 10% goat serum for 1 h at room temperature with primary or without (control) rabbit antibodies against *SLC5A5*/NIS (SAB2102220, Sigma) or *SLC26A4*/PENDRIN (MBS9215961, MyBioSource). The cells were washed and then stained for 30 min with secondary F(ab′)2 goat anti-rabbit (Invitrogen, A21246) at a 1:100 dilution and CD45 Krome orange (Beckman Coulter, A96416) at a 1:20 dilution at room temperature. Other experiments included CD14 FITC staining (BD Pharmingen, 555397) to identify monocytes. Cells were washed, resuspended in IsoFlow sheath fluid (Beckman Coulter), and then loaded onto BD FACSCanto II where 25,000 events were collected in the lymphocyte gate. The resulting data and the median fluorescence intensity (MFI) were analyzed utilizing FlowJo software.

### Detection of Thyroid Hormones *via* Immunoassay

To detect T_4_ or T_3_ hormones, 5 × 10^6^ leukocytes were washed and resuspended in 800 µL of complete RPMI or supplemented DMEM F-12 media (DMEM F-12 with 50 U/mL penicillin, 50 µg/mL streptomycin, non-essential amino acids, and MEM vitamin solution) (Gibco). The cells were incubated with or without native human TG (609312, Sigma) at a concentration of 20 µg/mL for 3 days. Supernatants from the cell culture were then loaded onto the Vitros ECiQ Immunodiagnostics instrument (Ortho molecular diagnostics) and assayed with Vitros reagents (total T_4_: 874468; total T_3_: 1322528; free T_4_: 1387000; free T_3_: 1315589).

### Statistical Analysis

Analysis and graphs/plots of all data were performed in GraphPad prism and Microsoft Excel software using two-tailed *t*-test assuming equal variance. Levels of significance *p* < 0.05 and *p* < 0.005 are presented as * and **, respectively.

## Results

### Human Leukocytes Express and Regulate Thyroid-Related Compounds

**Figure 1:**
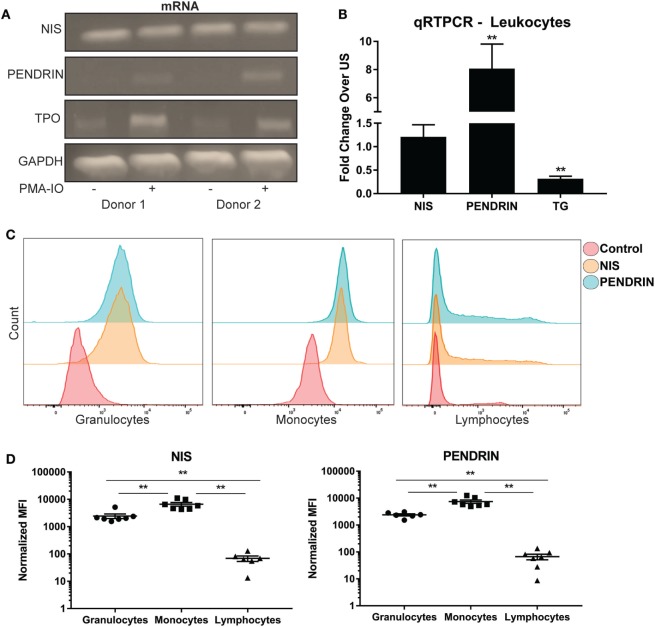
Human immune cells express and regulate iodide transporters, thyroglobulin, and thyroid peroxidase. **(A)** Leukocytes were left unstimulated (US) (−) or activated (+) with PMA and ionomycin for 18 h, total RNA was extracted, and then the cDNA from samples was amplified with PCR. Shown is DNA gel electrophoresis image of two representative donors. **(B)** Leukocytes mRNA was amplified using real-time quantitative PCR, and then each gene was normalized to internal B2M control. Fold increase over US controls was graphed ± SEM of nine independent donors. **(C)** Leukocytes were stained with no antibody (control), NIS, or PENDRIN antibodies. The cells were then stained with secondary Alexa-647 and primary-conjugated CD45 Krome-orange antibodies. Leukocyte subsets (granulocytes, monocytes, and lymphocytes) were gated on based on CD45 and side-scatter characteristics. The gated subset histograms are shown as cell counts and antibody staining intensity representative of six donors. **(D)** Median fluorescent intensity (MFI) values from panel **(C)** were subtracted from background staining and then graphed ± SEM of six to seven independent donors.

Previous work illustrated the ability of leukocytes to concentrate radio iodide (28). However, it is unclear how iodide is transported into immune cells or if leukocytes differentially express one or both of the known iodide transporters. To this end, human leukocytes were extracted from different donors, and then transcript levels of thyroid-related molecules were amplified with reverse transcription PCR. DNA gel electrophoresis analysis illustrated that leukocytes expressed expected size transcripts of NIS, and PENDRIN (Figure 1A). Activation of leukocytes with PMA and ionomycin (PMA-IO) induced a substantial increase of mRNA levels of PENDRIN but not NIS (Figure 1A). qPCR with TaqMan probes confirmed no significant change in NIS expression, but approximately eightfold increase of PENDRIN mRNA upon activation of leukocytes (Figure 1B). Although we could not find validated TaqMan probes that could amplify TPO, gel analysis revealed expected size TPO transcripts that were increased when leukocytes were activated (Figure 1A). Next, TG transcripts have been previously found in peripheral blood lymphocytes but it is not clear whether cellular activation regulates the mRNA expression of this protein (45). Since leukocytes upregulated PENDRIN and TPO, we sought to investigate the expression of the large dimeric protein TG during activation of leukocytes. Accordingly, mRNA analysis confirmed the expression of TG in leukocytes, but that activation significantly reduced its mRNA expression (Figure 1B).

To determine if NIS or PENDRIN are differentially expressed within peripheral blood leukocyte subsets, we utilized flow cytometric analysis of leukocytes probed with antibodies against NIS or PENDRIN. Leukocyte subpopulations were separated based on side scatter and CD45 intensity (Figure S1A in Supplementary Material), and then levels of iodide transporters were examined. NIS and PENDRIN were expressed in all leukocyte populations with the strongest expression detected in granulocyte and monocyte populations and minimal expression on lymphocytes (Figure 1C). Quantitative MFI analysis showed minor but non-significant MFI increase over background on lymphocytes, but strong and significant expression on granulocytes and monocytes (Figure 1D). Altogether, these results demonstrate that leukocytes, particularly monocytes and granulocytes, express and regulate thyroid-related compounds.

### Iodide Induces Human Leukocytes to Undergo Transcriptional Modification in Immunity-Related Genes

**Figure 2:**
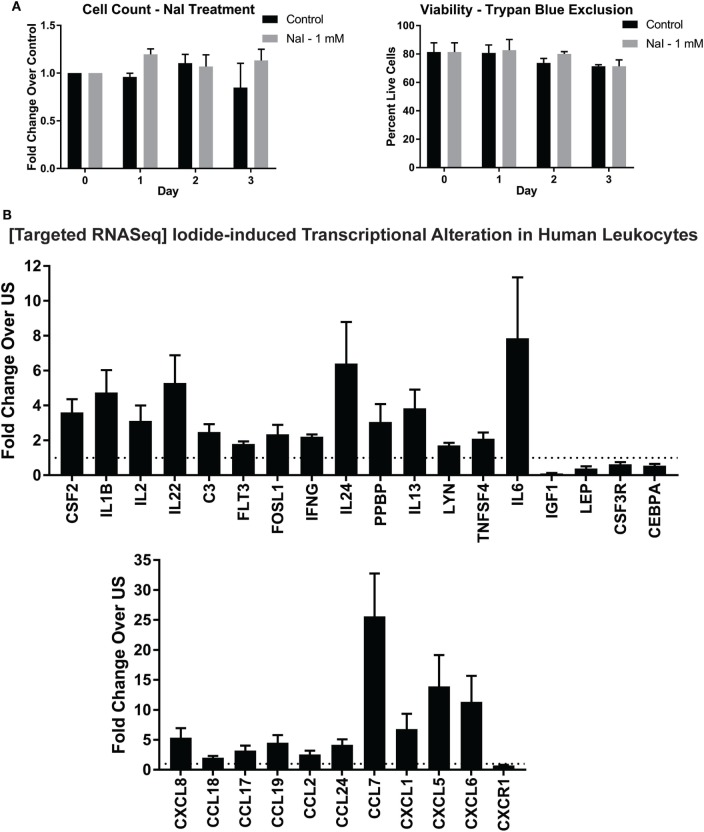
Targeted RNASeq analysis of iodide-treated leukocytes *via* next-generation sequencing. **(A)** Viability analysis of iodide-treated leukocytes—5 × 10^6^ leukocytes were left untreated (control—PBS) or incubated with 1 mM NaI for up to 3 days. Cell counts and viability utilizing trypan blue exclusion were determined with TC20 automated cell counter. **(B)** Targeted RNASeq—leukocytes were left unstimulated with PBS or incubated with 1 mM NaI for 48 h, total RNA was extracted, and then targeted RNASeq libraries were created for a total of 475 genes. The libraries were indexed (multiplexed) and then loaded onto Illumina MiSeq sequencer. The data were de-multiplexed, and unique molecular tags were identified utilizing Qiagen’s RNASeq bioinformatics software. Total molecular tag counts were normalized to counts of 10 housekeeping genes and then quantified based on fold expression relative to each untreated control. Average fold increases were obtained from 5 to 10 independent donors. Genes that were significantly increased or decreased (*p* < 0.05) were selected and displayed in bar graph ± SEM of at least five independent donors. See Table S1 in Supplementary Material for quantifications and *p* values.

No studies thus far have determined if iodine or iodide could directly affect the transcriptome of immune cells. Since treatment with Lugol’s solution containing iodine/iodide could alter transcriptional changes in breast cancer cell lines, and leukocytes expressed iodide transporters (Figure 1), we asked if iodide could alter immunity-related transcriptional events in human immune cells (38). We first investigated whether iodide had any toxic effects on human leukocytes. Preceding studies in breast cancer cell lines found no toxic effects using 1 mM of Lugol’s iodine solution (38). To confirm these studies in primary immune cells, we incubated leukocytes with 1 mM of NaI for 3 days. Overall, we found no significant changes in total cell counts or viability with iodide treatment (Figure 2A).

To determine if iodide treatment had any effect on the transcriptional activity of human leukocytes, we utilized NGS in the context of targeted RNASeq. This method allows quantification of hundreds of immunity-related genes per sample. To this end, leukocytes were incubated for 2 days with 1 mM NaI or PBS control, and then RNA was extracted. NGS library preparations were created, and then control or NaI-treated leukocytes were screened for changes in 475 inflammation and immunity genes. We found that immune cells treated with iodide had significant changes in total 29 genes with 24 being upregulated (Figure 2B; Table S1 in Supplementary Material). Although all transcriptional changes presented were statistically significant, IL6 and pro-platelet basic protein had a *p* value trending close to significance (0.085 and 0.076, respectively) due to variability despite the observed fold increase in all treatment groups (Table S1 in Supplementary Material). Genes that were upregulated included modulators that could affect survival or proliferation such as IL2, IL24, and CSF2. Interestingly, we observed increased expression of various cytokines that are considered to be pro- and/or anti inflammatory including IFNγ, IL6, IL1β, and IL13 (Figure 2B). However, chemokines comprised a significant fraction of the total altered genes and showed overall the highest fold increase with CCL7, CXCL5, and CXCL6 transcripts increasing by more than 10-fold in the iodide-treated groups (Figure 2B; Table S1 in Supplementary Material). Other transcriptional increases observed were in SRC kinase LYN, and complement C3. Next, iodide treatment significantly reduced the expression of both insulin growth factor-1 and leptin indicating the potential for iodide to affect hormonal balance. Altogether, our data illustrate the molecular immunomodulatory effects of iodide on human immune cells.

### Immune Cells Increase Cytokine Secretion after Iodide Treatment

**Figure 3:**
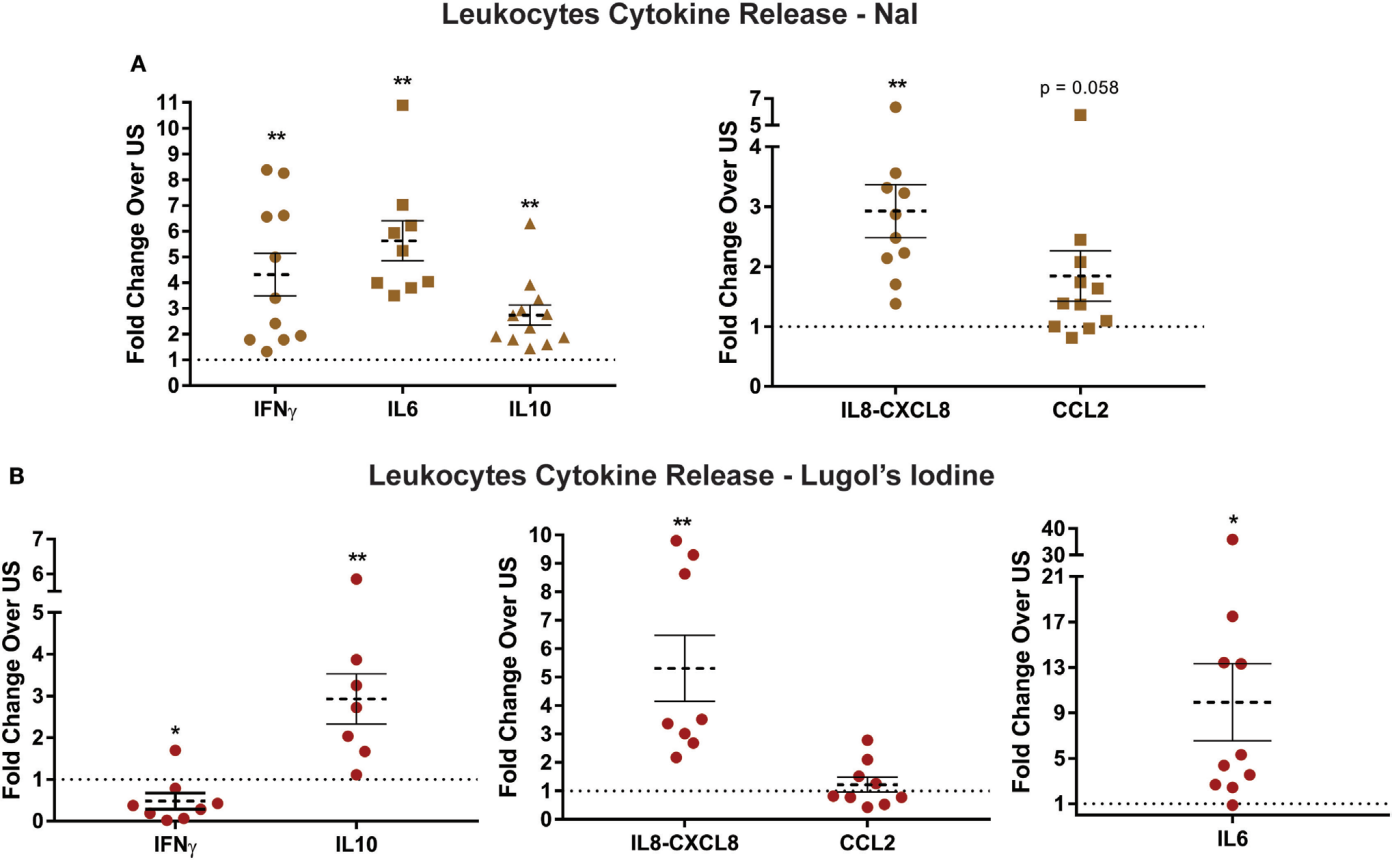
Increased release of cytokines and chemokines by iodide-treated leukocytes. **(A)** 5 × 10^6^ leukocytes were left unstimulated (US) with PBS or treated with 1 mM NaI for 72 h. The supernatants were collected, and protein levels of cytokines/chemokines were analyzed utilizing enzyme-linked immunosorbent assay. **(B)** Same as in panel **(A)**, but instead leukocytes were incubated with 500 µM Lugol’s iodine for 72 h, and then the supernatant was analyzed for cytokine levels. Detected ranges for cytokine secretion are (IFNγ: 5–1,005 pg/mL, IL6: 10–2,500 pg/mL, IL10: 26–482 pg/mL, IL8-CXCL8: 0.3–230 ng/mL, and CCL2: 15–65 ng/mL). Cytokine concentrations were normalized based on fold changes over each US pair and was then averaged and graphed as fold increase ± SEM of at least seven donors.

Basal level elevation of chemokine and cytokine transcripts after iodide treatment suggested that immune cells may consequently increase protein release. Accordingly, we examined the quantity of select cytokines comparing control and iodide-treated leukocytes. Basal levels of pro- and anti-inflammatory cytokines including IFNγ, IL6, and IL10 were substantially increased in the presence of iodide in cell culture 3 days after treatment (Figure 3A). IL6 production was the most affected with more than fivefold average increase in protein release (Figure 3A). Similarly, chemokines IL8 (CXCL8) and CCL2 were elevated after iodide treatment. However, changes in CCL2 were variable with 3 out of 11 donors showing no change or slight reduction in cytokines levels (Figure 3A).

**Figure 4:**
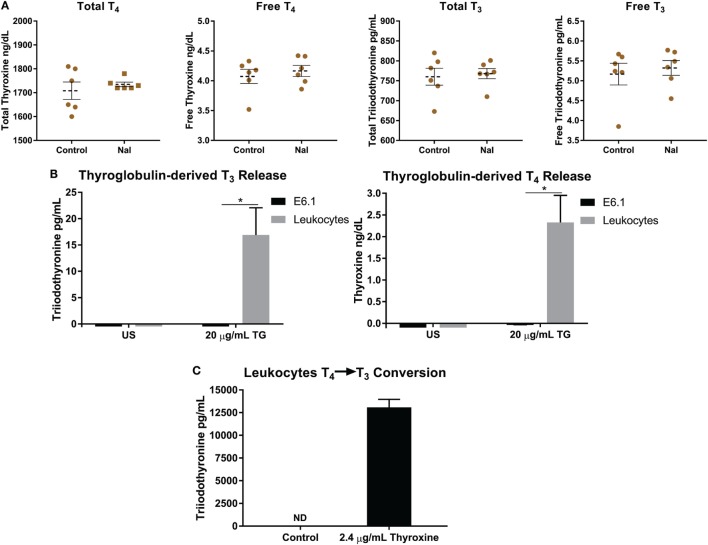
Thyroglobulin (TG) is utilized by leukocytes to increase levels of thyroid hormones. **(A)** 5 × 10^6^ leukocytes were left unstimulated with PBS or treated with 1 mM NaI for 72 h, and then levels of total and free thyroid hormones in media were detected utilizing ECiQ diagnostic immunoassay instrument. Shown are averaged concentrations ± SEM of six donors. **(B)** 5 × 10^6^ leukocytes were incubated with TG derived from human thyroid tissue at a concentration of 20 µg/mL for 3 days in serum free media, and then levels thyroid hormones (T_4_ and T_3_) in media were detected utilizing ECiQ diagnostic immunoassay instrument. The data were averaged after background subtraction of TG signal. Shown are averaged concentrations ± SEM of six donors. **(C)** Same as in panel **(B)**, but instead leukocytes were incubated with thyroxine (T_4_) at 2.4 µg/mL for 2 days in serum free media. Levels of T_3_ were analyzed utilizing diagnostic immunoassay instrument ECiQ. The data were averaged after background subtraction of T_4_ signals. Data shown represent averaged quantifications ± SEM of six donors.

To determine if other forms of iodine could affect basal cytokine release, we incubated leukocytes with 500 µM of Lugol’s iodine. That is, instead of NaI, the cells would be incubated with a mixture of KI and I_2_. Similar to our observations with NaI, leukocytes exposed to Lugol’s iodine had even greater increase of protein release in IL6, IL10, and CXCL8 (Figure 3B). However, we were surprised to find that Lugol’s iodine significantly reduced release of IFNγ (0.47-fold) compared with controls. Next, though not statistically significant, CCL2 secretion displayed a similar pattern to NaI-treated cells wherein five out of nine donors had reduced levels (Figure 4B). The subtle differences between NaI and Lugol’s can be explained by the distinct iodine-derivatives between the two treatments. These data suggest that iodine could affect the functional activity of human immune cells.

### Iodide-Induced Cytokine and Transcriptional Alteration Is Not due to *De Novo* Thyroid Hormone Synthesis or Deiodination

Thyroid hormones are important for the metabolic function of all cells, but also carry the ability to affect the cytokine and chemokine profiles, phenotypes, and function of immune cells (20, 22, 23, 46). One possible reason for the iodide-induced elevation of mRNA and cytokine release in leukocytes would be an increase of *de novo* T_4_ synthesis and/or by the known ability of immune cells to deiodinate T_4_ present in media into T_3_ (18, 47–49). Therefore, we analyzed supernatants of NaI-pulsed leukocytes for possible fluctuations of thyroid hormones. Utilizing immunodiagnostics immunoassay ECiQ instrument, we could not detect any significant differences in free and total forms of thyroid hormones between controls and NaI-treated leukocytes (Figure 4A). Next, we have attempted to expose leukocytes to various experimental settings in an effort to expose potential *de novo* T_4_ synthesis and release. To this end, leukocytes were incubated in serum free media (to remove serum hormones) supplemented with amino acids with added vitamins and minerals along with 1 mM NaI and/or 500 µM Lugol’s solution. These experiments could not produce any detectable T_4_ or T_3_ in the supernatant (data not shown). In addition, stimulating leukocytes with PMA-IO or anti-TCR antibodies in the presence of NaI or Lugol’s iodine could not produce detectable levels of T_4_ or T_3_ in the supernatant (data not shown). These observations indicate that the increased specific transcription and cytokine release by Lugol’s iodine and NaI-treated leukocytes is not driven by new synthesis of T_4_ or T_3_.

### Immune Cells Utilize TG to Increase the Levels of Thyroid Hormones T_4_ and T_3_

During an iodine deficiency TG is substantially increased in the blood causing it to potentially interact with cells and tissues (50–52). Thus, far no reports have demonstrated if TG could also be utilized by immune or other cells types. Since thyroid hormones affect the immune system, we asked if human leukocytes could utilize thyroid-derived TG to increase surrounding levels of T_4_ and T_3_ (20, 22, 23, 53, 54). To this end, we incubated leukocytes with TG for 3 days in serum free media, to avoid interference with hormones present in serum, and then levels of T_4_ and T_3_ were detected. Interestingly, leukocytes incubated with TG released substantial amounts of T_4_ and T_3_ wherein no hormones were detected in untreated cells (Figure 4B). We were surprised to observe that this effect was not carried on to the Jurkat T cell line suggesting a non T cell-mediated mechanism (Figure 4B). These results suggest that a leukocyte subset, likely phagocytes, could endocytose TG, release T_4_ and then produce T_3_ by deiodination. Next, the thyroid is estimated to contain hormones consisting of 80–90% T_4_ and approximately 10–20% T_3_ stored mostly within TG (15–17). Therefore, we assume that the increased levels of T_3_ after TG incubation is due to T_4_ deiodination by immune cells. To confirm that human leukocytes could deiodinate T_4_, the cells were instead given T_4_ and then levels of T_3_ were analyzed. Confirming previously reported observations, leukocytes could produce significant amounts of T_3_ *via* deiodination (Figure 4C) (47–49, 55). Our data reflect the interaction between human immune cells and TG, and that immune cells could potentially raise levels of T_4_ and T_3_ in tissues and peripheral blood.

## Discussion

**Figure 5:**
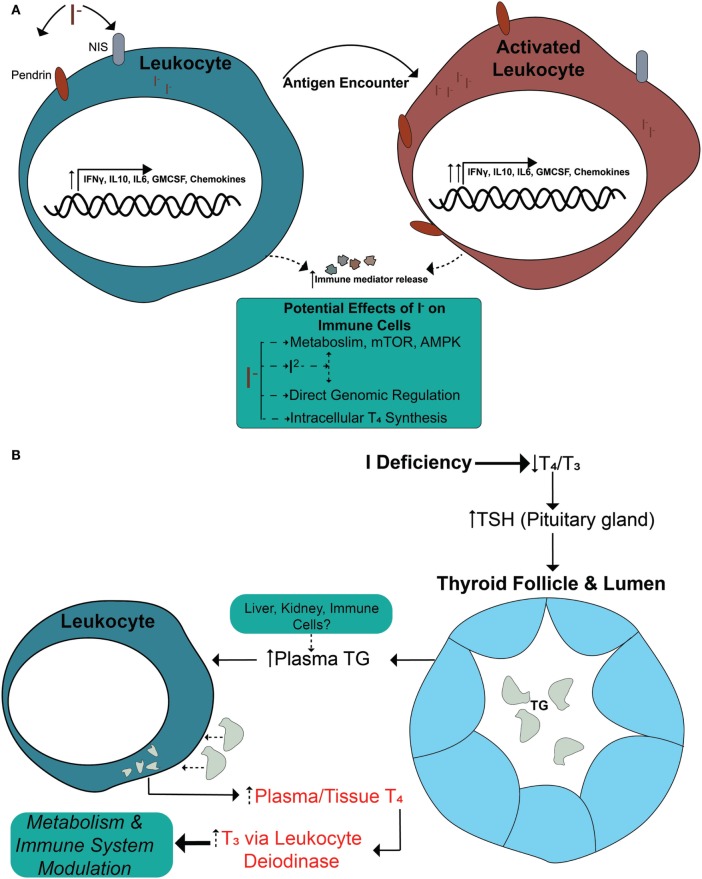
Current model—immune cells regulate their function *via* iodide and increase levels of thyroid hormones by processing thyroid-derived thyroglobulin (TG). **(A)** Immune cells express surface iodide transporters (NIS and PENDRIN) that are upregulated during cellular activation. The cells are able to accumulate iodide that could alter the transcription of multiple immune mediators and possibly other genes. The changes are functional since the higher mRNA levels correlate with increased cytokine release at the basal state. The effect is systemic and is not polarized to either pro- or anti-inflammatory genes. During an immune response, the presence of sufficient amounts of iodide allows for a “primed” state of cells that are ready to proliferate upon activation. The effects of iodide on immune cells could have an impact during early conception wherein immune cells could release more factors to support blood vessel and optimal pregnancy. **(B)** In an iodine deficiency state, TSH is secreted to command an increase of mostly T_4_ and some T_3_ by the thyroid. The thyroid responds by elevating NIS and PENDRIN surface expression and the production of more TG, some of which is released in the blood stream. Increased severity of iodine deficiency raises TG levels in the blood accordingly. Based on our findings, we propose that leukocytes could uptake TG from the blood or tissues and release T_4_, which will eventually raise T_3_ levels by deiodinase activity. This has a systemic effect since it increases levels of hormones to local tissues and/or in the blood for increased metabolism. Released thyroid hormones are also known to affect the immune system by enhancing cytokine expression and altering phenotypes of immune cells.

In this study, we have identified immunomodulatory effects of iodide on human immune cells. We have demonstrated that, in the presence of iodide, human immune cells undergo specific molecular changes in certain cytokines and chemokines, and that this effect is translated into higher protein release (Figure 5A). These events are not caused by increased thyroid hormone synthesis, but instead by an unclear iodide specific mechanism. However, our investigation into the possibility of thyroid hormone synthesis by leukocytes revealed the capability of immune cells to process TG and release the thyroid hormones T_4_ and T_3_ (Figures 4B and 5B).

Analysis of iodide receptor expression on leukocyte subsets demonstrated that phagocytes, monocytes, and granulocytes harbor the highest expression of iodide transporters (Figures 1C,D). Earlier studies reported the interplay between leukocyte myeloperoxidase and H_2_O_2_, with the halides chloride and iodide (56–58). The authors illustrate the antibacterial effect of myeloperoxidase mediated iodination, which was more effective in killing bacteria than chloride. The observed increase in PENDRIN post activation of immune cells, a transporter specific for both chloride and iodide, suggests that both ions are important for the killing mechanisms of monocytes and granulocytes (Figures 1A,B) (57). Therefore, it is likely that the mechanism of iodide intracellular transport would be used by phagocytes to clear infections. The idea of iodide receptor regulation is not new as the effect of hormones and cytokines on iodide transporters have been previously illustrated (59–61). We cannot confirm if the increase in PENDRIN and TPO in our experimental setting was due to intracellular activation by PMA-IO or secondary to cytokine release by leukocytes post activation. The increase of PENDRIN and TPO expression post PMA-IO stimulation implies that leukocytes may require increased iodide uptake when activated during an immune response. This hypothesis is strengthened by our results where increased iodide substantially raises the secretion levels of multiple cytokines. Surprisingly, lymphocytes exhibited relatively minimal expression of NIS and PENDRIN (Figures 1C,D). However, this does not imply that lymphocytes are unresponsive to iodide as it has been clearly demonstrated that exposure to iodide produces increases in immunoglobulin synthesis by lymphocytes with even lower doses of iodide used in this study (62).

Assessment of immunity-related genes *via* targeted RNASeq in iodide-treated leukocytes revealed significant transcriptional changes in 29 out 475 analyzed genes. The transcriptional changes observed in leukocytes were not polarized but instead constituted a mix of cytokines that were pro- and anti-inflammatory including IFNγ, IL6, and IL13. Differentiation and survival factors such as IL2, IL24, and CSF2 were also increased *via* iodide stimulation. The effects of iodide, however, were striking with regards to chemokines and their receptors which constituted 11 out of the 29 altered genes (Figure 2B). The most substantial transcriptional fold changes were associated with chemokines CCL7 (22.5), CXCL5 (13.9), and CXCL6 (11.3) (Figure 2B; Table S1 in Supplementary Material). The concept that iodine/iodide causes transcriptional changes is not new as iodine stimulation was shown to induce multiple transcriptional changes in human breast cancer and trophoblastic cell lines (38, 63). It is therefore very likely that non-immunity transcriptional changes would be occurring in leukocytes in the presence of iodine/iodide. Similar to breast cancer and trophoblastic cell lines, these changes may include genes associated with estrogen metabolism, cyclins, and transcription factors (38, 63). Next, although we have analyzed a short list of cytokines *via* ELISA, the cytokine release profile was not skewed to either pro- or anti-inflammatory, but instead seems to be systemic thereby mimicking the observed transcriptional changes. It is unclear how iodide induces these changes, but it is likely a combination of mechanisms such as an enhancement of metabolic pathways or intracellular conversion of iodide into iodine (Figure 5A) (64, 65). We also suggest the possibility of iodine influencing transcription by directly or indirectly activating transcription factors, or by altering enhancer/promoter sequences in the genome. In this case, iodine would mirror a similar role to zinc which could activate transcription factors to increase cytokine expression as well as interact directly with DNA (66–68).

One limitation of our study is that we have utilized a mixed leukocyte population and therefore it is not clear which cell population is contributing to the transcriptional changes. Moreover, some cell types (i.e., granulocytes) are short lived and may not represent the transcriptional changes presented in this study. Similarly, monocytes adhere to cell culture plates and could be lost during the extraction phase from culture plates. To this end, we compared leukocyte populations on the day of extraction and 2 days after cell culture *via* flow cytometry. There were no significant differences on the percentages of monocyte or lymphocyte populations 2 days after cell culture (Figures S1B,C and Table S2 in Supplementary Material). However, the monocyte population had increased side scatter but maintained exclusive expression of CD14 as in day 0. Treatment of cells for 2 days with 1 mM NaI did not cause significant changes to the percentages of leukocyte subsets. Analysis of the granulocyte fraction, however, illustrated significant reduction in cell percentage 2 days post cell culture. Therefore, our analyses in this study represent lymphocytes, monocytes, and a relatively small number of granulocytes. Importantly, in our experimental setting, the leukocyte populations utilized in targeted RNASeq had similar viabilities and were overall comparable in percentage to initial day of extraction (Figure 2A; Figures S1B,C in Supplementary Material).

An objective of this study was to determine if leukocytes could produce *de novo* thyroid hormones that could potentially affect systemic and/or local hormone levels. Previous experiments with radioiodine provided an insight on synthesis of low intracellular T_4_ by cardiomyocytes (29). The authors argue that the low level of intracellular T_4_ would only affect the synthesizing cardiomyocyte and not surrounding cells (29). Likewise, the presence of “thyroxine-like” compounds in ^131^I-pulsed leukocytes suggested the possibility of thyroid hormone synthesis by extrathyroidal tissues (28). Under our experimental settings, we could not detect any increase of T_4_ or T_3_ when the cells were incubated with 1 mM of NaI for 72 h in complete media (Figure 4A). Likewise, addition of Lugol’s iodine or NaI to leukocytes in amino acid and vitamin supplemented serum free media with or without cellular activation did not yield any detectable levels of T_4_ or T_3_ in the supernatant (data not shown). We cannot, however, exclude the possibility of intracellular *de novo* T_4_ synthesis by leukocytes or very low levels of T_4_ below the sensitivity range of our immunoassay instrument. On the other hand, we observed a marked increase of T_4_ and T_3_ in culture media after leukocytes were incubated with TG (Figure 4B). These hormones were already present on TG since it was derived from human thyroid that under normal conditions should contain 80–90% T_4_. These observations indicate that immune cells could implement the last two out of the three following steps performed by the thyroid glands for hormone synthesis: (1) organification of iodine into TG, (2) endocytosis of TG containing thyroid hormones, and (3) release of T_4_ and some T_3_ into peripheral blood. Next, during the process of T_4_ release, leukocytes were able to deiodinate TG-derived T_4_ into T_3_ thereby increasing levels of the active thyroid hormone T_3_ (Figure 4C). The interaction of immune cells with TG is physiologically relevant since TG is present in the blood and is significantly increased during an iodine deficiency (51, 52). Altogether, these results shed light into the long-sought question of whether tissues other than the thyroid could influence blood or local levels of T_4_ and demonstrate that at least immune cells could potentially affect blood thyroid hormone levels.

We have utilized in this study a concentration of 1 mM NaI (~125 μg/mL of iodide) that was non-toxic to primary human immune cells. In fact, though not significant, NaI-treated cells had slightly higher cell number counts (Figure 2A). Moreover, this dose was non-toxic when used on human breast cancer cell lines (38). For these reasons, we opted to use this concentration for targeted RNA sequencing and functional studies in primary human immune cells. Observed population plasma levels of inorganic iodine (i.e., non hormonal iodine) are relatively low with total iodine in the range of 50–130 µg/L and an inorganic iodide range of 5–15 µg/L (69). In comparison with plasma iodide levels, thyroidal cells are exposed to 50- to 400-fold more of inorganic iodide (69). It is highly feasible that tissue resident immune cells, including intestinal or thymic cells, could be exposed to substantially higher levels of iodine relative to levels observed in plasma (37). Blood iodine levels reflect recent ingested iodine intakes and are not typically utilized for determining long-term iodine sufficiency status due partially to the kinetics of inorganic iodide metabolism where it is either absorbed and stored by tissues or excreted rapidly by the kidneys (69, 70). This is observed in persons with sufficient iodine levels where typically an iodine urinary loading test shows excretion of 90% or more of the ingested iodine within 24 h and much less in iodine deficient populations (71, 72). In fact, studies focusing on assessment of long-term levels of iodine status suggest biomarkers such as TG or urinary iodine as the more sensitive markers for iodine levels (50–52, 73, 74). Ingested iodine is therefore either rapidly absorbed by the thyroid and tissues expressing iodine transporters that are widely distributed or is excreted by the urine in quantities inversely correlated with whole-body sufficiency (28, 69, 72). Above all, optimal whole-body sufficiency levels of iodine are unknown, and the current RDA recommendations are provided primarily as preventative for goiter formation (2, 69). Future clinical investigations are needed to determine the safe daily quantity of iodine necessary for extrathyroidal tissue sufficiency.

Next, iodine is required for a successful healthy pregnancy, and its loss leads to miscarriages, reproductive failures, abnormal brain development, and congenital hypothyroidism (3, 9, 10). Epidemiological studies and surveys by the World Health Organization demonstrate that iodine deficiencies are occurring worldwide including in women of reproductive age (2, 5, 7, 75, 76). Recent findings by the National Health and Nutrition Examination Survey found that up to 35% of women in reproductive age have an iodine insufficiency (75, 76). The issue of iodine sufficiency during pregnancy is compounded as a result for higher iodine intake requirements during pregnancy and lactation (2, 7). Based on our findings and the role of the immune system in regulating the process of pregnancy, we suggest further investigation between immune cell dysfunction in female reproductive organs and the possibility of iodine deficiency in women with reproductive failures of unknown etiology.

In conclusion, we have presented evidence for the immunomodulatory effects of iodide on human peripheral blood immune cells. Iodide alters the transcriptional immune signature of these cells and induces stronger cytokine and chemokine responses. Accordingly, iodine/iodide levels that optimally saturate the cells should therefore enhance the immune system and improve trafficking, clearance of infections, and support the process of reproduction. Finally, we identify immune cells as a potential source of extrathyroidal thyroid hormones capable of performing functions typically known to be specific to the thyroid glands.

## Ethics Statement

The research presented here was performed according to the principles indicated in the declaration of Helsinki. The consent and documentation process associated with donors utilized in this study were approved by the IRB for Rosalind Franklin University of Medicine and Science.

## Author Contributions

MB conceived, designed, and performed the experiments; interpreted the data; and wrote the manuscript with input from all other authors. SD, JK-K, AG-S, and KB contributed to study design and data analysis.

## Conflict of Interest Statement

The authors declare that the research was conducted in the absence of any commercial or financial relationships that could be construed as a potential conflict of interest.

## Supplementary Material

Figure S1 Leukocyte populations and viabilities post iodide treatment. **(A)** Leukocytes were stained with primary-conjugated CD45 Krome-orange antibodies. Leukocyte subsets (granulocytes, monocytes, and lymphocytes) were gated on based on CD45 and side-scatter characteristics. **(B)** Leukocytes were stained with CD45 Krome orange and CD14 FITC on the day of isolation or after 2 days of culture on 12-well culture dishes with or without 1 mM NaI. Shown is a representative donor with color coded scatter plots illustrating granulocytes, monocytes, and lymphocytes. **(C)** Leukocytes population percentages from panel **(B)** were averaged and graphed. Data shown represent averaged quantifications ± SD of six donors. See Table S2 in Supplementary Material for quantifications and *p* values.

## References

[1] Rousset B Dupuy C Miot F Dumont J Thyroid hormone synthesis and secretion. In: De Groot LJ Chrousos G Dungan K Feingold KR Grossman A Hershman JM Koch C Korbonits M McLachlan R New M Purnell J Rebar R Singer F Vinik A, editors. Endotext. (Chap. 2), South Dartmouth, MA (2000). p. 2–3. Available from: http://www.thyroidmanager.org/wp-content/uploads/chapters/chapter-2-thyroid-hormone-synthesis-and-secretion.pdf

[2] Benoist BD Andersson M Egli I Takkouche B Allen H Iodine Status Worldwide. Geneva: World Health Organization (2004).

[3] Verheesen RH Schweitzer CM. Iodine deficiency, more than cretinism and goiter. Med Hypotheses (2008) 71(5):645–8. 10.1016/j.mehy.2008.06.020 18703293

[4] Ahad F Ganie SA. Iodine, Iodine metabolism and Iodine deficiency disorders revisited. Indian J Endocrinol Metab (2010) 14(1):13–7. 21448409 PMC3063534

[5] Dillon JC Milliez J. Reproductive failure in women living in iodine deficient areas of West Africa. BJOG (2000) 107(5):631–6. 10.1111/j.1471-0528.2000.tb13305.x 10826578

[6] Perez-Lopez FR. Iodine and thyroid hormones during pregnancy and postpartum. Gynecol Endocrinol (2007) 23(7):414–28. 10.1080/09513590701464092 17701774

[7] Zimmermann MB. The effects of iodine deficiency in pregnancy and infancy. Paediatr Perinat Epidemiol (2012) 26(Suppl 1):108–17. 10.1111/j.1365-3016.2012.01275.x 22742605

[8] Sukkhojaiwaratkul D Mahachoklertwattana P Poomthavorn P Panburana P Chailurkit LO Khlairit P Effects of maternal iodine supplementation during pregnancy and lactation on iodine status and neonatal thyroid-stimulating hormone. J Perinatol (2014) 34(8):594–8. 10.1038/jp.2014.62 24743135

[9] Kapil U. Health consequences of iodine deficiency. Sultan Qaboos Univ Med J (2007) 7(3):267–72. 21748117 PMC3074887

[10] Pharoah P Buttfield IH Hetzel BS Neurological damage to the fetus resulting from severe iodine deficiency during pregnancy. Int J Epidemiol (2012) 41(3):589–92. 10.1093/ije/dys070 22586135

[11] Laurberg P Andersen SL Keep an eye on iodine and the thyroid and save the brain. Horm Res Paediatr (2014) 81(6):361–2. 10.1159/000360700 24853111

[12] Wilders-Truschnig MM Warnkross H Leb G Langsteger W Eber O Tiran A The effect of treatment with levothyroxine or iodine on thyroid size and thyroid growth stimulating immunoglobulins in endemic goitre patients. Clin Endocrinol (Oxf) (1993) 39(3):281–6. 10.1111/j.1365-2265.1993.tb02367.x 7900936

[13] Dumont JE Ermans AM Maenhaut C Coppee F Stanbury JB Large goitre as a maladaptation to iodine deficiency. Clin Endocrinol (Oxf) (1995) 43(1):1–10. 10.1111/j.1365-2265.1995.tb01886.x 7641399

[14] Spitzweg C Heufelder AE Morris JC Thyroid iodine transport. Thyroid (2000) 10(4):321–30. 10.1089/thy.2000.10.321 10807060

[15] Barrett EJ Synthesis of thyroid hormones. In: Boron WF Boulpaep EL, editors. Medical Physiology – A Cellular and Molecular Approach. 2nd ed Philadelphia, PA: Saunders Elsevier (2012).

[16] Laurberg P. Mechanisms governing the relative proportions of thyroxine and 3,5,3’-triiodothyronine in thyroid secretion. Metabolism (1984) 33(4):379–92. 10.1016/0026-0495(84)90203-8 6369072

[17] Mortoglou A Candiloros H. The serum triiodothyronine to thyroxine (T3/T4) ratio in various thyroid disorders and after levothyroxine replacement therapy. Hormones (Athens) (2004) 3(2):120–6. 10.14310/horm.2002.11120 16982586

[18] Bianco AC Kim BW. Deiodinases: implications of the local control of thyroid hormone action. J Clin Invest (2006) 116(10):2571–9. 10.1172/JCI29812 17016550 PMC1578599

[19] Balazs C Leovey A Szabo M Bako G. Stimulating effect of triiodothyronine on cell-mediated immunity. Eur J Clin Pharmacol (1980) 17(1):19–23. 10.1007/BF00561672 6966220

[20] Klecha AJ Genaro AM Gorelik G Barreiro Arcos ML Silberman DM Schuman M Integrative study of hypothalamus-pituitary-thyroid-immune system interaction: thyroid hormone-mediated modulation of lymphocyte activity through the protein kinase C signaling pathway. J Endocrinol (2006) 189(1):45–55. 10.1677/joe.1.06137 16614380

[21] Hodkinson CF Simpson EE Beattie JH O’Connor JM Campbell DJ Strain JJ Preliminary evidence of immune function modulation by thyroid hormones in healthy men and women aged 55–70 years. J Endocrinol (2009) 202(1):55–63. 10.1677/JOE-08-0488 19398496

[22] De Vito P Incerpi S Pedersen JZ Luly P Davis FB Davis PJ. Thyroid hormones as modulators of immune activities at the cellular level. Thyroid (2011) 21(8):879–90. 10.1089/thy.2010.0429 21745103

[23] Alamino VA Montesinos MM Rabinovich GA Pellizas CG The thyroid hormone triiodothyronine reinvigorates dendritic cells and potentiates anti-tumor immunity. Oncoimmunology (2016) 5(1):e1064579 10.1080/2162402X.2015.1064579 26942081 PMC4760281

[24] Bagriacik EU Zhou Q Wang HC Klein JR. Rapid and transient reduction in circulating thyroid hormones following systemic antigen priming: implications for functional collaboration between dendritic cells and thyroid. Cell Immunol (2001) 212(2):92–100. 10.1006/cimm.2001.1846 11748925

[25] Csaba G Pallinger E Thyrotropic hormone (TSH) regulation of triiodothyronine (T(3)) concentration in immune cells. Inflamm Res (2009) 58(3):151–4. 10.1007/s00011-008-8076-8 19205847

[26] Obregon MJ Mallol J Escobar del Rey F Morreale de Escobar G. Presence of L-thyroxine and 3,5,3’-triiodo-L-thyronine in tissues from thyroidectomized rats. Endocrinology (1981) 109(3):908–13. 10.1210/endo-109-3-908 7262025

[27] Nagao H Imazu T Hayashi H Takahashi K Minato K. Influence of thyroidectomy on thyroxine metabolism and turnover rate in rats. J Endocrinol (2011) 210(1):117–23. 10.1530/JOE-10-0484 21478227

[28] Stolc V Stimulation of iodoproteins and thyroxine formation in human leukocytes by phagocytosis. Biochem Biophys Res Commun (1971) 45(1):159–66. 10.1016/0006-291X(71)90064-7 5139919

[29] Meischl C Buermans HP Hazes T Zuidwijk MJ Musters RJ Boer C H9c2 cardiomyoblasts produce thyroid hormone. Am J Physiol Cell Physiol (2008) 294(5):C1227–33. 10.1152/ajpcell.00328.2007 18322142

[30] Ullberg S Ewaldsson B Distribution of radio-iodine studied by whole-body autoradiography. Acta Radiol Ther Phys Biol (1964) 2:24–32. 10.3109/02841866409134127 14153759

[31] Spitzweg C Joba W Eisenmenger W Heufelder AE. Analysis of human sodium iodide symporter gene expression in extrathyroidal tissues and cloning of its complementary deoxyribonucleic acids from salivary gland, mammary gland, and gastric mucosa. J Clin Endocrinol Metab (1998) 83(5):1746–51. 10.1210/jcem.83.5.4839 9589686

[32] Wapnir IL van de Rijn M Nowels K Amenta PS Walton K Montgomery K Immunohistochemical profile of the sodium/iodide symporter in thyroid, breast, and other carcinomas using high density tissue microarrays and conventional sections. J Clin Endocrinol Metab (2003) 88(4):1880–8. 10.1210/jc.2002-021544 12679487

[33] Di Cosmo C Fanelli G Tonacchera M Ferrarini E Dimida A Agretti P The sodium-iodide symporter expression in placental tissue at different gestational age: an immunohistochemical study. Clin Endocrinol (Oxf) (2006) 65(4):544–8. 10.1111/j.1365-2265.2006.02577.x 16984250

[34] Catalano RD Critchley HO Heikinheimo O Baird DT Hapangama D Sherwin JR Mifepristone induced progesterone withdrawal reveals novel regulatory pathways in human endometrium. Mol Hum Reprod (2007) 13(9):641–54. 10.1093/molehr/gam021 17584828

[35] Marani L Venturi S Masala R Role of iodine in delayed immune response. Isr J Med Sci (1985) 21(10):864. 4077481

[36] Marani L Venturi S. [Iodine and delayed immunity]. Minerva Med (1986) 77(19):805–9. 3714096

[37] Venturi S Venturi M Iodine, thymus, and immunity. Nutrition (2009) 25(9):977–9. 10.1016/j.nut.2009.06.002 19647627

[38] Stoddard FR II Brooks AD Eskin BA Johannes GJ. Iodine alters gene expression in the MCF7 breast cancer cell line: evidence for an anti-estrogen effect of iodine. Int J Med Sci (2008) 5(4):189–96. 10.7150/ijms.5.189 18645607 PMC2452979

[39] Venturi S Venturi M Iodine in evolution of salivary glands and in oral health. Nutr Health (2009) 20(2):119–34. 10.1177/026010600902000204 19835108

[40] Riesco-Eizaguirre G Leoni SG Mendiola M Estevez-Cebrero MA Gallego MI Redondo A NIS mediates iodide uptake in the female reproductive tract and is a poor prognostic factor in ovarian cancer. J Clin Endocrinol Metab (2014) 99(7):E1199–208. 10.1210/jc.2013-4249 24708099

[41] Mor G Cardenas I Abrahams V Guller S. Inflammation and pregnancy: the role of the immune system at the implantation site. Ann N Y Acad Sci (2011) 1221:80–7. 10.1111/j.1749-6632.2010.05938.x 21401634 PMC3078586

[42] Beaman KD Ntrivalas E Mallers TM Jaiswal MK Kwak-Kim J Gilman-Sachs A. Immune etiology of recurrent pregnancy loss and its diagnosis. Am J Reprod Immunol (2012) 67(4):319–25. 10.1111/j.1600-0897.2012.01118.x 22380608

[43] Beaman KD Dambaeva S Katara GK Kulshrestha A Gilman-Sachs A. The immune response in pregnancy and in cancer is active and supportive of placental and tumor cell growth not their destruction. Gynecol Oncol (2017) 145(3):476–80. 10.1016/j.ygyno.2017.04.019 28477880

[44] Bowen JM Chamley L Keelan JA Mitchell MD. Cytokines of the placenta and extra-placental membranes: roles and regulation during human pregnancy and parturition. Placenta (2002) 23(4):257–73. 10.1053/plac.2001.0782 11969336

[45] Amakawa M Kato R Kameko F Maruyama M Tajiri J. Thyroglobulin mRNA expression in peripheral blood lymphocytes of healthy subjects and patients with thyroid disease. Clin Chim Acta (2008) 390(1–2):97–103. 10.1016/j.cca.2008.01.007 18243140

[46] Davis PJ Glinsky GV Lin HY Mousa SA. Actions of thyroid hormone analogues on chemokines. J Immunol Res (2016) 2016:3147671. 10.1155/2016/3147671 27493972 PMC4967430

[47] Holm AC Lemarchand-Beraud T Scazziga BR Cuttelod S. Human lymphocyte binding and deiodination of thyroid hormones in relation to thyroid function. Acta Endocrinol (Copenh) (1975) 80(4):642–56. 171899 10.1530/acta.0.0800642

[48] Smekens L Golstein J Vanhaelst L. Measurement of thyroxine conversion to triiodothyronine using human lymphocytes. A useful and simple laboratory technique. J Endocrinol Invest (1983) 6(2):113–7. 10.1007/BF03350582 6863848

[49] Bianco AC Nunes MT Marone MS Correa PH. Conversion of thyroxine (T4) to T3 and rT3 in human leucocyte suspension: its application to clinical investigation. Endocrinol Exp (1985) 19(1):53–61. 3921342

[50] Pezzino V Vigneri R Squatrito S Filetti S Camus M Polosa P. Increased serum thyroglobulin levels in patients with nontoxic goiter. J Clin Endocrinol Metab (1978) 46(4):653–7. 10.1210/jcem-46-4-653 755050

[51] Vejbjerg P Knudsen N Perrild H Laurberg P Carle A Pedersen IB Thyroglobulin as a marker of iodine nutrition status in the general population. Eur J Endocrinol (2009) 161(3):475–81. 10.1530/EJE-09-0262 19556382

[52] Bath SC Pop VJ Furmidge-Owen VL Broeren MA Rayman MP Thyroglobulin as a functional biomarker of iodine status in a cohort study of pregnant women in the United Kingdom. Thyroid (2017) 27(3):426–33. 10.1089/thy.2016.0322 27762729 PMC5337401

[53] Chen Y. Effect of thyroxine on the immune response of mice in vivo and in vitro. Immunol Commun (1980) 9(3):260–76. 10.3109/08820138009065999 6995277

[54] Paavonen T. Enhancement of human B lymphocyte differentiation in vitro by thyroid hormone. Scand J Immunol (1982) 15(2):211–5. 10.1111/j.1365-3083.1982.tb00640.x 6980446

[55] Woeber KA. L-triiodothyronine and L-reverse-triiodothyronine generation in the human polymorphonuclear leukocyte. J Clin Invest (1978) 62(3):577–84. 10.1172/JCI109163 690186 PMC371802

[56] Klebanoff SJ. Iodination of bacteria: a bactericidal mechanism. J Exp Med (1967) 126(6):1063–78. 10.1084/jem.126.6.1063 4964565 PMC2138423

[57] Klebanoff SJ. Myeloperoxidase-halide-hydrogen peroxide antibacterial system. J Bacteriol (1968) 95(6):2131–8. 4970226 10.1128/jb.95.6.2131-2138.1968 PMC315145

[58] Klebanoff SJ Kettle AJ Rosen H Winterbourn CC Nauseef WM. Myeloperoxidase: a front-line defender against phagocytosed microorganisms. J Leukoc Biol (2013) 93(2):185–98. 10.1189/jlb.0712349 23066164 PMC3545676

[59] Uyttersprot N Pelgrims N Carrasco N Gervy C Maenhaut C Dumont JE Moderate doses of iodide in vivo inhibit cell proliferation and the expression of thyroperoxidase and Na+/I- symporter mRNAs in dog thyroid. Mol Cell Endocrinol (1997) 131(2):195–203. 10.1016/S0303-7207(97)00108-1 9296378

[60] Spitzweg C Joba W Morris JC Heufelder AE Regulation of sodium iodide symporter gene expression in FRTL-5 rat thyroid cells. Thyroid (1999) 9(8):821–30. 10.1089/thy.1999.9.821 10482376

[61] Dohan O De la Vieja A Paroder V Riedel C Artani M Reed M The sodium/iodide symporter (NIS): characterization, regulation, and medical significance. Endocr Rev (2003) 24(1):48–77. 10.1210/er.2001-0029 12588808

[62] Weetman AP McGregor AM Campbell H Lazarus JH Ibbertson HK Hall R. Iodide enhances IgG synthesis by human peripheral blood lymphocytes in vitro. Acta Endocrinol (Copenh) (1983) 103(2):210–5. 6858554 10.1530/acta.0.1030210

[63] Olivo-Vidal ZE Rodriguez RC Arroyo-Helguera O. Iodine affects differentiation and migration process in trophoblastic cells. Biol Trace Elem Res (2016) 169(2):180–8. 10.1007/s12011-015-0433-1 26152853

[64] Carling D Thornton C Woods A Sanders MJ. AMP-activated protein kinase: new regulation, new roles? Biochem J (2012) 445(1):11–27. 10.1042/BJ20120546 22702974

[65] Chapman NM Chi H mTOR signaling, Tregs and immune modulation. Immunotherapy (2014) 6(12):1295–311. 10.2217/imt.14.84 25524385 PMC4291176

[66] Aydemir TB Liuzzi JP McClellan S Cousins RJ. Zinc transporter ZIP8 (SLC39A8) and zinc influence IFN-gamma expression in activated human T cells. J Leukoc Biol (2009) 86(2):337–48. 10.1189/jlb.1208759 19401385 PMC2726764

[67] Klug A. The discovery of zinc fingers and their applications in gene regulation and genome manipulation. Annu Rev Biochem (2010) 79:213–31. 10.1146/annurev-biochem-010909-095056 20192761

[68] Foster M Samman S. Zinc and regulation of inflammatory cytokines: implications for cardiometabolic disease. Nutrients (2012) 4(7):676–94. 10.3390/nu4070676 22852057 PMC3407988

[69] Risher JF Keith S Iodine and Inorganic Iodides: Human Health Aspects. Geneva: World Health Organization (2009).

[70] Vought RL London WT Lutwak L Dublin TD Reliability of estimates of serum inorganic iodine and daily fecal and urinary iodine excretion from single casual specimens. J Clin Endocrinol Metab (1963) 23:1218–28. 10.1210/jcem-23-12-1218 14087601

[71] Pearce EN Andersson M Zimmermann MB. Global iodine nutrition: where do we stand in 2013? Thyroid (2013) 23(5):523–8. 10.1089/thy.2013.0128 23472655

[72] Haap M Roth HJ Huber T Dittmann H Wahl R. Urinary iodine: comparison of a simple method for its determination in microplates with measurement by inductively-coupled plasma mass spectrometry. Sci Rep (2017) 7:39835. 10.1038/srep39835 28045077 PMC5206638

[73] Ma ZF Skeaff SA. Thyroglobulin as a biomarker of iodine deficiency: a review. Thyroid (2014) 24(8):1195–209. 10.1089/thy.2014.0052 24762031 PMC4106385

[74] Ershow AG Goodman G Coates PM Swanson CA. Research needs for assessing iodine intake, iodine status, and the effects of maternal iodine supplementation. Am J Clin Nutr (2016) 104(Suppl 3):941S–9S. 10.3945/ajcn.116.134858 27534640 PMC5004498

[75] Caldwell KL Makhmudov A Ely E Jones RL Wang RY Iodine status of the U.S. population, National Health and Nutrition Examination Survey, 2005–2006 and 2007–2008. Thyroid (2011) 21(4):419–27. 10.1089/thy.2010.0077 21323596

[76] Leung AM Pearce EN Braverman LE. Iodine nutrition in pregnancy and lactation. Endocrinol Metab Clin North Am (2011) 40(4):765–77. 10.1016/j.ecl.2011.08.001 22108279 PMC3266621

