# Supplementary figures and images for: A Role for Iodide and Thyroglobulin in Modulating the Function of Human Immune Cells
Source: Front Immunol. 2017 Nov 15;8:1573. doi: 10.3389/fimmu.2017.01573 (PMC5694785; doi:10.3389/fimmu.2017.01573)

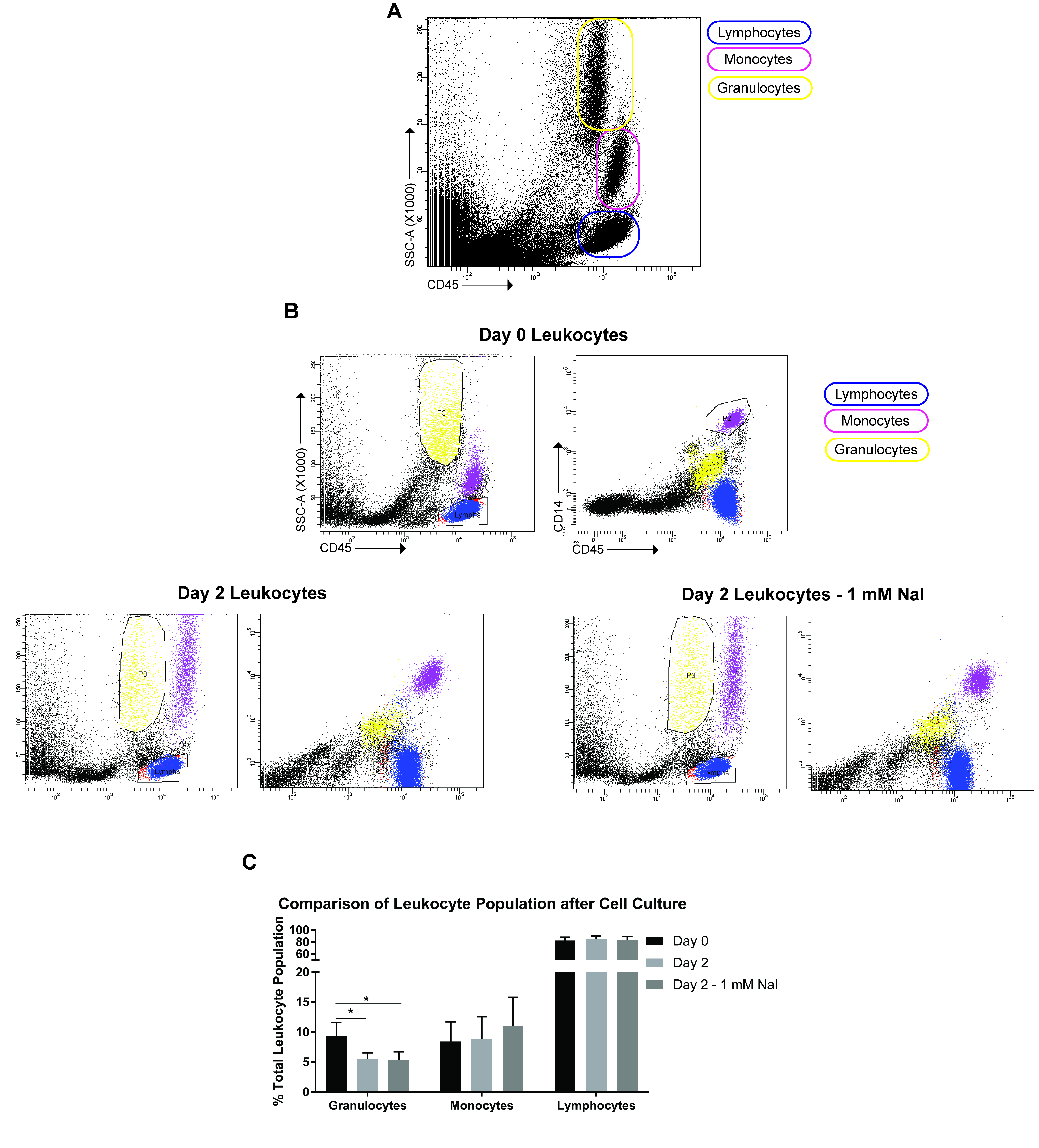

Supplement: Figure S1 — Leukocyte populations and viabilities post iodide treatment. (A) Leukocytes were stained with primary-conjugated CD45 Krome-orange antibodies. Leukocyte subsets (granulocytes, monocytes, and lymphocytes) were gated on based on CD45 and side-scatter characteristics. (B) Leukocytes were stained with CD45 Krome orange and CD14 FITC on the day of isolation or after 2 days of culture on 12-well culture dishes with or without 1 mM NaI. Shown is a representative donor with color coded scatter plots illustrating granulocytes, monocytes, and lymphocytes. (C) Leukocytes population percentages from panel (B) were averaged and graphed. Data shown represent averaged quantifications ± SD of six donors. See Table S2 in Supplementary Material for quantifications and p values. [file image_1.tif]
